# Immunological Landscape of HER-2 Positive Breast Cancer

**DOI:** 10.3390/cancers14133167

**Published:** 2022-06-28

**Authors:** Santiago Moragon, Cristina Hernando, Maria Teresa Martinez-Martinez, Marta Tapia, Belen Ortega-Morillo, Ana Lluch, Begoña Bermejo, Juan Miguel Cejalvo

**Affiliations:** 1 Department of Medical Oncology, INCLIVA Biomedical Research Institute, University of Valencia, 46010 Valencia, Spain; moragon_san@gva.es (S.M.); hernando_cri@gva.es (C.H.); martinez_mtemar@gva.es (M.T.M.-M.); tapia_marces@gva.es (M.T.); ortega_bel@gva.es (B.O.-M.); lluch_ana@gva.es (A.L.); bermejo_beg@gva.es (B.B.); 2 Instituto de Salud Carlos III, CIBERONC (Centro De Investigacion Biomedica En Red De Cancer), 28220 Madrid, Spain

**Keywords:** HER2+ breast cancer, immune checkpoint blockade, anti-HER2 vaccines, tumour-infiltrating lymphocytes, PD-L1

## Abstract

**Simple Summary:**

The aim of this work is to elucidate the role of immune response in HER2+ breast cancer. By describing the principal components of the tumour microenvironment and its interaction with the known and novel anti-HER2 targeted therapies, this review summarizes the ways immune modulators could be implemented in clinical practice.

**Abstract:**

Understanding the biological aspects of immune response in HER2+ breast cancer is crucial to implementing new treatment strategies in these patients. It is well known that anti-HER2 therapy has improved survival in this population, yet a substantial percentage may relapse, creating a need within the scientific community to uncover resistance mechanisms and determine how to overcome them. This systematic review indicates the immunological mechanisms through which trastuzumab and other agents target cancer cells, also outlining the main trials studying immune checkpoint blockade. Finally, we report on anti-HER2 vaccines and include a figure exemplifying their mechanisms of action.

## 1. Introduction

Treatment and prognosis of Human epidermal growth factor receptor 2-positive (HER2+) breast cancer patients have been successfully improved by implementing novel HER-2 targeted agents. Antibody-drug conjugates such as ado-trastuzumab emtasine (T-DM1) and trastuzumab-Deruxtecan (T-DXd) have enhanced the life expectancy in this patient subgroup [1,2].

Nevertheless, dysregulation and suppression of the immune system lead to tumour cell survival and disease progression. Reinstating immune response is mandatory to achieve anti-tumour activity [3].

Widespread use of immune checkpoint inhibitors (ICIs), such as Programmed cell death protein (PD)-1, Programmed death-ligand 1 (PD-L1) and Cytotoxic T-Lymphocyte Antigen 4 (CTLA4) inhibitors have recently changed the comprehension and treatment of solid tumours, representing a new paradigm in oncology.

However, implementing the use of immunotherapy in breast cancer patients remains a challenge, as breast carcinoma is generally considered a moderately immunogenic tumour. HER2+ and triple-negative breast cancer (TNBC) are known to be more immunogenic than luminal type, and are associated with higher rates of cell proliferation and genomic instability, as well as augmented tumour-infiltrating lymphocytes (TIL) levels [4]. Indeed, tumour infiltration by T cells has been linked to favourable prognosis in different solid tumours, including HER2+ and TNBC [5,6].

Tumours with higher proliferation rates are classed as genetically unstable; they exhibit higher levels of clonal heterogeneity, leading to increased cell death and more production of neoantigens, which are in turn exposed to available antigen-presenting cells (APC), resulting in T and B cell recruitment and activation [6].

Moreover, HER2 protein is considered a tumour-associated antigen (TAA) that is targeted by trastuzumab, causing anti-tumour effect through apoptosis, cell arrest induction, antibody-dependent cell-mediated cytotoxicity (ADCC), inhibition of HER2 extracellular domain shedding, and inhibition of downstream signaling [7].

Tumour microenvironment (TME) contains several immunosuppressive molecules such as vascular endothelial growth factor (VEGF), cyclooxygenase (COX)-2, interleukin (IL)-6, IL-10, transforming growth-factor-beta (TGF- β), stemcell factor-1, and macrophage colony-stimulating factor (M-CSF) which all inhibit antigen presentation. Furthermore, activated CD4+ helper and CD8+ cytotoxic lymphocytes (CTLs) stimulate type 1 immunity, which leads to anti-tumour activity by secreting tumour necrosis factor (TNF)- α, interferon (IFN)- γ, and other cytotoxins [8,9,10].

In the clinical setting, multiple ongoing clinical trials are evaluating different strategies involving, among others, ICIs and TME modulators.

This review aims to describe the main molecular mechanisms of immune response in HER2+ BC patients, the mechanism of action of anti-HER2 agents, and the clinical activity of novel drugs such as ICIs and anti-HER2 vaccines. The TME, its components, and its interactions with anti-HER2 agents will also be discussed.

## 2. Tumour Microenvironment

**Figure 1:**
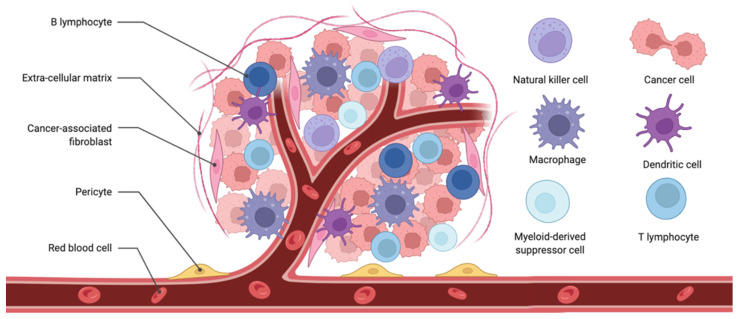
Scheme of tumour microenvironment.

The TME is composed of the immune system cells such as T and B cells, DCs, Myeloid-derived suppressor cells (MDSCs), and tumour-associated macrophages (TAMs), which form a complicated network of fibroblasts, blood vessels, lymphatics, and cancer cells themselves (Figure 1).

The interaction between these and the diverse cell population within the TME influences tumour resistance, progression, and metastasis [11,12,13].

Immune evasion is mainly due to poor or non-expression of surface antigens or co-stimulatory molecules, down-regulation of major histocompatibility complex (MHC) I or T-cell signaling defects. Furthermore, TME infiltration by inhibitory immune cells such as regulatory T cells (Tregs), MDSCs, or inhibitory cytokines (TGF-β or IL-10) can also enhance immune evasion [14,15].

Activated HER2 oncogene might also have a role in regulating infiltrating tumour cells in the tumour mutational burden (TMB). Triulzi et al. (2019) performed a transcriptome profiling analysis of archival formalin-fixed paraffin embedded (FFPE) tumour blocks from HER2+ samples treated with adjuvant trastuzumab, finding a correlation between trastuzumab-sensitive tumours and higher expression of chemokines such as CCL2, in contrast to trastuzumab non-sensitive tumours. In an in vivo model, trastuzumab was dependent on CCL2 expression levels and monocytes infiltration in the TME. Interestingly, they also found that ER expression could block in vitro CCL2 production by inhibiting the NF-kB pathway [16].

Modulation of the TME and angiogenesis is an unmet clinical need in HER2+ BC. Indoleamine-pyrrole 2,3-dioxygenase 1 (IDO1) is an immunosuppressive enzyme responsible for tryptophan catabolism in the TME. IDO1 leads to a higher rate of tryptophan conversion and depletion. This induces cell cycle arrest of cytotoxic T cells and their subsequent depletion [17].

In a subset analysis of breast tumour samples, up to 40% of the HER2+ samples had high IDO levels. IDO overexpression was seen in higher levels in TNBC and HER2+ tumours than ER+/HER2- tumours (OR = 0.29 (0.08–1.08, *p* = 0.06) and OR = 0.76 (0.33–1.72, *p* = 0.51)), respectively [18]. IDO1-targeting drugs are considered to enhance immune checkpoint blockers’ efficacy. A variety of ongoing clinical trials are currently assessing IDO-1 inhibitors in combination with ICIs in different solid tumours [19]. Moreover, Toll-like receptors (TLR) have also been shown to be an important link between innate and adaptive immunity through their presence in DCs. TLR-2 has been shown to drive trastuzumab-mediated cytotoxicity against HER2+ breast tumour cells. TLR agonists are currently being tested in combination with HER2-directed vaccines (NCT02276300) [20]. TILS, TMB, PD-L1 expression, microsatellite instability (MSI), and the novel tertiary lymphoid structures are possible biomarkers which together could stimulate an immune response in HER-2+ BC patients.

### 2.1. Tumour-Infiltrating Lymphocytes

Tumour-Infiltrating Lymphocytes (TILs) consist of lymphocytic cells, Natural killer (NK) cells, DCs, and macrophages surrounding and invading the tumour stroma. They are a crucial component of the TME in different types of solid tumours, nevertheless, the percentage of stroma invaded by TILs differs across breast cancer subtypes. As already mentioned, more aggressive subtypes such as HER2+ BC and TNBC have a higher TIL rate and, therefore suggestive of a more favourable response to immunotherapy [21].

Soberino et al. (2019) described the influence and prognostic value of TILs infiltrating in the (neo)adjuvant and advanced HER2+ disease (Table 1) [4].

As observed in the CLEOPATRA and APHINITY trials, there is a positive association between high TILs (cut-off values > 20%) and improved prognosis in trastuzumab-pertuzumab-containing regimens in both advanced and early settings. This event, by contrast, is not experienced in the advanced setting with trastuzumab or lapatinib alone [48].

Furthermore, TIL level could be a prognostic marker to identify patients needing a shorter duration of anti-HER2 therapy in the early stages. The ShortHER adjuvant trial compared 9 weeks versus 1-year of adjuvant trastuzumab in addition to chemotherapy. In a retrospective analysis, for patients with TILs < 20%, the distant disease-free survival (DFS) was improved only after full 1-year duration of therapy (*p* = 0.021), while for patients with TILs ≥ 20% the distant DFS rate was improved in the short arm versus the long arm (*p* = 0.015) [49].

In addition, TILs were also tested in samples from the BIG 02-98 adjuvant phase III trial, which compared an anthracycline-only regimen (doxorubicin followed by cyclophosphamide, methotrexate, and fluorouracil (CMF) or doxorubicin plus cyclophosphamide followed by CMF) versus chemotherapy combining doxorubicin and docetaxel, regardless of BC subtype. TILs increase by 10% was associated with a reduced risk of relapse and death in hormone receptor (HR)-negative/HER2- patients regardless of a chemotherapy regimen.

In HER2-positive BC, there was a significant interaction between increasing tumour and stromal lymphocytic infiltration by 10% increments and DFS benefit from an anthracycline-only regimen (*p* = 0.08). According to the authors, this could have been mediated by higher accumulative doses of anthracyclines inducing immunogenic cell death, and secondly, by the immunosuppressive effect of corticosteroids in the docetaxel arm. Trastuzumab-containing treatments were not assessed [50]. Identifying patients at higher risk of relapse based on baseline TIL levels is therefore useful for early disease detection.

TILs were also tested in both PANACEA and KATE2 trials in assessing checkpoint inhibitors in advanced disease, and only patients with high stromal TIL levels responded to ICIs [46,47].

### 2.2. Tertiary Lymphoid Structures

Tertiary lymphoid structures (TLSs) are ectopic lymphoid organs developing in non-lymphoid tissues where chronic inflammatory signals are highly expressed. They are composed of a T cell-rich zone with mature DCs juxtaposing a B cell follicle with germinal centre characteristics and are surrounded by plasma cells. TLSs are predominantly found in the tumoural stroma and in the invasive margin of the stroma than in the core of the tumour. The presence of follicular dendritic cells (FDCs) and germinal centres within TLSs along with CD4+ and CD8+ and high endothelial venules (HEVs) surrounding them makes them resemble secondary lymphoid organs, which is where efficient adaptive immune response against cancer occurs [51,52]. Vanhersecke et al. (2021) recently demonstrated that TLSs might be a novel valuable predictive biomarker of response to immunotherapy independently of PD-L1 expression [53]. In the context of BC, Prabhakaran et al. (2017) predicted that the presence of TLSs was linked to better survival and partial complete response to neoadjuvant chemotherapy across high-grade, HR-negative, HER2+ BC tumours [54]. Moreover, the clinicopathological association and prognostic role of TLSs were examined across 248 BC samples by Liu et al. TLSs were associated with HR-negative, HER2+ tumours along with c-kit expression. Across HER2+ BC, a better DFS was found in tumours presenting high TLSs expression, and the combined TLS and TIL status was an independent favourable factor associated with DFS (*p* = 0.006) [55]. Finally, a meta-analysis assessed the prognostic value of TLSs and their correlation with clinicopathological features, gene expression, and survival in all BC subtypes. A higher density of TLSs was reported to be significantly correlated with better DFS in all subtypes (HR = 0.40, 95% CI: 0.17−0.93, *p* < 0.001) [56].

### 2.3. Tumour Mutational Burden

The mutational load indicates the frequency of certain mutations within tumour genes, and an increased load leads to antigenicity and immunogenicity, suggesting longer survival for patients treated with ICIs harboring a higher TMB [57].

Among all BC subtypes, TNBC has a higher TMB than oestrogen receptor (ER)-positive or HER2+ BC [58,59].

Ee Park et al. performed a whole exome sequencing (WES) on 46 tumour samples from metastatic HER2+ patients, of which 30% were ER+ and 20% were progesterone receptor (PR) positive. High TMB was defined as >100 nonsynonymous single nucleotide variant (SNV) mutations. Median overall survival (OS) differed significantly between low and high TMB status (44.9 months vs. 85.8 months, respectively, *p* = 0.016). In the multivariate analysis, TMB was the only independent prognostic factor for metastatic overall survival (*p* = 0.049). According to this study, TMB might be a good predictive biomarker for OS in HER2+ metastatic breast cancer (mBC) patients [60].

### 2.4. PD-L1 Expression

Immune response is partially regulated by the interaction of PD-L1 antigen on tumour cells with PD-1 on the immune cells, thereby suppressing T-cell activity in the tumour. PD-L1 expression is currently being used as a predictive biomarker in some solid tumours such as non-small cell lung cancer (NSCLC) or metastatic urothelial cancer, representing a change in clinical practice [61,62].

PD-L1 expression is not homogeneous across all breast tumours. Higher expression of this biomarker is generally associated with higher TIL infiltrate, and probably also associated with more aggressive subtypes such as TNBC and HER2+ BC [63].

A study performed on HER2+ BC samples from early stage disease showed that high expression of CD8+ and PD-L1+ cells was associated with longer survival, suggesting a role in checkpoint inhibition to be explored in this subgroup [64]. In the aforementioned PANACEA trial, the 15% of patients who had PD-L1+ tumours had a partial response to trastuzumab and pembrolizumab, which contrasted with the lack of response in PD-L1- patients. Moreover, only the subpopulation of patients with PD-L1+ (measured by gene expression) in the KATE2 trial benefited from T-DM1 added to atezolizumab [46,47].

### 2.5. Microsatellite Instability

Understanding the cell cycle and genomic instability has paved the discovery of novel therapeutic agents against solid tumours. Indeed, cyclin-dependent kinases (Cdk) 4 and 6 inhibitors in combination with endocrine treatment have improved survival in HR+/Her2- MBC in first and second-line settings [65]. Moreover, mismatch-repair deficiency (dMMR) tumours exhibit higher TIL count and a hypermutated phenotype (>10 mutations per megabase) suggesting that these would be more suitable for immunotherapy. In fact, ICIs are approved for any tumour harbouring MSI [66]. The prevalence of MSI-H/dMMR in BC is less than 2% overall. According to a report by S. Cheng et al. (2020), only 5 out of 31 patients with MSI-H/dMMR were HER2+ BC patients, and only 2 were HER2 enriched by intrinsic subtype testing [67]. Given the lack of clinical data regarding the efficacy of pembrolizumab in BC harbouring MSI-H/dMMR, this biomarker requires further assessment in clinical trials.

## 3. Immune-Mediated Effects by HER2- Targeted Therapies

Dual anti-HER2 blockade with directed monoclonal antibodies (mAbs) such as trastuzumab and pertuzumab has demonstrated strong clinical benefit for patients, and changed the paradigm of HER2+ breast cancer [42,68].

Trastuzumab is an IgG1 recombinant humanized monoclonal Ab (mAb) against the extracellular domain of HER2, which induces cell-cycle arrest in the G1 phase and inhibits phosphatidylinositol 3-kinase (PI3K)-Akt signalling, antibody-dependent cellular cytotoxicity (ADCC), complement-mediated cytotoxicity (CDC) and inhibition of angiogenesis [8,12,69].

ADCC is promoted by binding the crystallizable (Fc) region of a mAb to the crystallizable fragment receptor (FcR) of the NK cell (specifically CD16 or FcyRIII), thereby releasing cytokines (such as granzymes, perforin, IFN-a or TNF-b) which will, in turn, induce tumour cell death. Moreover, FcyR polymorphisms have been proven to alter mAb-mediated therapy in cancer. FcyRIIa and FcyRIIIa are receptors present in immune cells, which are activated by binding to the Fc portion of trastuzumab. Different studies support the evidence that FcγRIIA-H/H131 and FcγRIIIA-V/V158 are associated with clinical response [70,71,72], while some others suggest that FcγRIIA-H/H, but not FcγRIIIA-V/V158, is associated with the efficacy of trastuzumab [73]. Nevertheless, the prediction of benefits from trastuzumab has been related to its interaction with not only innate immunity but also adaptive immunity, TIL count, and immune-related signatures. In consequence, T cells are crucial for tumour reduction by anti HER2+ antibody. In an in vivo experiment, T cell-deficient mice showed a rapid relapse of the tumour. Moreover, T cell infiltration (mostly CD8+ cells) was detected in tumour tissue of mice treated with anti-HER2 antibody [74].

In line with this, Takada et al. (2018) evaluated immune biomarkers from biopsy specimens from 30 patients who had received treatment for metastatic HER2+ disease with trastuzumab, pertuzumab and docetaxel. A higher CD8+/FOXP3 ratio was indicative of a higher benefit in progression-free survival (PFS) (*p* = 0.0027) [75]. In addition, Song et al. (2020) performed a co-culture between HER2+ BC cell lines and CD4+ T cells, comparing viability when treated with or without trastuzumab. The viability of HER2 + cancer cells decreased significantly when exposed to trastuzumab and T-cells, compared to cancer cells exposed to trastuzumab without T-cells (*p* = 0.01), suggesting trastuzumab may trigger immune-mediated cytotoxicity [76]. Pertuzumab is a humanized IgG1 mAb with a complementary function to trastuzumab as it binds to a different epitope of the HER2 extracellular domain (domain II). It also induces ADCC and CDC pathways, blocks HER2-HER3 dimerization in the presence of heregulin (HRG), and therefore downregulates the PI3K-Akt pathway, inducing an anti-trophic effect [77]. Both trastuzumab and pertuzumab equally activate ADCC against HER2+ tumour cells, but no increase in its potency is seen when both agents are used in combination. Conversely, targeting in vitro HER2+ BC cells with either one or the other agent has only minor effects in CDC, whereas combination therapy neutralizes complement regulatory proteins (such as CD46, CD55, and CD59) followed by an antibody-induced C3 opsonization and killing of tumour cells [78,79].

New molecules have been successfully developed in recent years. Margetuximab is a chimeric anti-HER2 mAb with a modified Fc domain for improved binding to FcyRIIIa and lower binding to FcyRIIB [80]. Its Fc region mediates ADCC which is in turn carried out by NK cells and monocytes. In fact, in the SOPHIA phase 3 trial comparing margetuximab vs. trastuzumab plus chemotherapy in an advanced HER2+ setting, patients carrying FcyRIIIa 158F allele had better clinical outcomes (median PFS):7 months vs. 5 months, HR 0.68; 95% CI 0.52–0.90; *p* = 0.005) [81].

Novel antibody-drug conjugates (ADC) are expected to achieve a breakthrough in HER2+ BC. T-DM1 is composed of trastuzumab and DM1, a microtubule-depolymerizing agent which facilitates DC functions and contributes to immune response [82].

The more recent T-DXd contains a topoisomerase inhibitor molecule as a payload. In contrast to T-DM1, it has a higher drug-to-antibody ratio (approximately 8 vs. 3 to 4), is a tumour-selective cleavable linker and is able to provoke a bystander anti-tumour effect, thereby allowing for a potent cytotoxic effect in nearby cells [83].

T-DXd has been associated with enhancing antitumour immunity in mice models by increasing the expression of MHC class I protein on cancer cells, upregulating the expression of DC markers (CD86+) and CD8+ cells in the TME. Furthermore, T- DXd in combination with either an anti-PD-1 or an anti-CTLA4 antibody has demonstrated enhanced antitumour activity in mouse models and in a phase Ib trial in combination with nivolumab with ORR up to 60% [84,85,86]. There might also be differences in terms of efficacy between these two ADCs. The recent phase III trial DESTINY-Breast03 trial compared the efficacy and safety of T-DXd vs. T-DM1 in previously treated HER2+ metastatic BC. The trial met its primary endpoint as the median PFS was not reached in the T-DXd arm, while in the T-DM1 arm was 6.8 (Hazard ratio (HR) for PFS was 0.28; IC 95%: 0.22–0.37) [87].

Zenocotuzumab (MCLA-128) is a novel bispecific IgG1 anti-HER2 Ab targeting HER2 and HER3 receptors under enhanced ADCC, thereby blocking HER2-HER3 heterodimerization and down-signaling the neuregulin/HER3 tumour-signalling pathway [88].

Fostering the interaction between immune cells and HER2+ cancer cells is another strategy to enhance immune response. The bispecific tribody [(HER2)_2_xCD16] redirects CD16-expressing γδ T cells and NK cells against HER2-expressing cells in order to enhance the cytotoxic anti-tumour effect [89]. Although this molecule showed no increase in objective anti-tumour response rate in a phase I/II trial, it did induce adaptive immune responses to both intracellular and extracellular domains of HER2 [90].

Cinrebafusp alfa (PRS-343) is a bispecific fusion protein which comprises a HER2-antibody and a unique 4-1BB (CD137) anticalin protein. CD137 provides co-stimulatory signals and activates cytotoxic effects of CD8+ T cells, as well as activating NK and DCs which further supports cytotoxic T cell activation [91]. Cinrebafusp alfa creates a bridge between CD137+ cells and HER2-expressing cells, causing immune cells to cluster around tumour cells, which leads to signal cascade and T cell activation. A phase I dose escalation study showed clinical benefit and safety of PRS-343 in HER2+ solid tumours [92]. There is also an ongoing phase 1b trial assessing the role of anti-PD-L1 blockade with atezolizumab added to cinrebafusp alfa in patients with HER2+ advanced solid tumours [93].

Runimotamab (BTRC4017A) a is HER2/anti-CD3 T-cell-dependent bispecific mAb considered to present both immunostimulatory and antineoplastic effects. By binding simultaneously to CD3-expressing T cells and HER2- expressing tumour cells it creates a bridge between these latter to potentiate the T-cell cytotoxic effect against HER2+ tumour cells. An ongoing phase I trial is currently assessing Runimotamab on locally advanced or metastatic HER2-expressing solid tumours (NCT03448042) [94]. Beyond bispecific mAbs, there are a few other mechanisms of targeting HER2 by exploiting the immune system. Cytokines such as IFN-y in combination with anti-HER2 therapy has been shown to have a synergistic effect in reducing tumour growth in in vivo *models* [95]. RO6874281 (Simlukafusp-α) is an immunocytokine comprising an antibody targeting fibroblast activation protein α (FAP) and an IL-2 variant lacking IL-2Rα binding, with retained IL-2Rβγ binding (thus expanding immune effector cells in the absence of regulatory T-cells activation). It has proven to be safe and clinically active in combination with atezolizumab and bevacizumab in patients with metastatic renal cell carcinoma [96]. It is currently being studied in a phase I trial as a single agent or in combination with trastuzumab or cetuximab [97] (NCT02627274) and in a phase II study in combination with atezolizumab plus chemotherapy [98] (NCT03386721).

Lapatinib is a dual HER2-HER1 intracellular tyrosine kinase inhibitor. It inhibits phosphorylation of the HER2 tyrosine domain, which prevents ubiquitination, inducing accumulation of HER2 on the cell membrane. By up-regulation of its cell surface receptor, lapatinib enhances trastuzumab-mediated ADCC [99,100,101]. Lapatinib also has the ability to modulate the TME as it promotes tumour infiltration by CD4 + CD8 + IFN-γ-producing T-cells through a Stat1 dependent pathway [102]. Immune activation gene signatures have been tested in the NeoALTTO, PAMELA, and CHER-LOB neoadjuvant trials (see Table 1). In the NeoALTTO trial, immune gene signatures were associated with higher pCR only in the combination arm (odds ratio, 2.1; 95% CI, 1.2–4.0; interaction test *p* = 0.01) [103]. In the PAMELA trial, stromal TILs on day 15 of treatment (but not at baseline) were independently associated with pCR [32].

In the CHER-LOB trial, immune gene signatures were significantly associated with pCR in a multivariate analysis adjusted for PAM50 subtypes [104]. Finally, low CD8+ stromal TILs in primary tumour predict inferior response to lapatinib vs. trastuzumab in the metastatic setting, according to TIL assessment from the phase III RCT MA.31, which randomized metastatic HER2+ patients to receive a taxane plus either trastuzumab or lapatinib [55].

Neratinib is a pan-HER tyrosine kinase inhibitor, which blocks downstream pathways counteracting trastuzumab resistance [105]. Unlike lapatinib, its role as an immune modulator is still unclear [106]. Tucatinib is a third-generation anti-HER2 tyrosine kinase inhibitor with heightened activity in the central nervous system. The HER2CLIMB study was the first randomised placebo-controlled trial to demonstrate a significant benefit in OS and PFS for patients with active central nervous system HER2+ disease [107]. Tucatinib has been shown to increase infiltration of NK cells, CD8 T-cells, IFN-γ, Ki67, and TIM-3 in in vivo murine models, suggesting that tucatinib could favourably modulate the TME favorably and synergise with ICIs [108].

## 4. Immune Therapies for HER2+ Breast Cancer

### 4.1. Immune Checkpoint Blockade for HER2+ BC

By considering TMB, PD-L1 expression, TLSs, and TILs as possible predictive biomarkers for immunotherapy, we can affirm that there is a strong rationale for checkpoint inhibition in HER2+ BC, however, results to date have not been very promising.

There are only a few published RCTs assessing PD-1/PD-L1 blockade in HER2+ BC patients. Table 2 shows the published clinical trials with ICIs.

The randomized phase II KATE2 trial assessed adding atezolizumab to T-DM1 in HER2+ MBC patients after progression to a taxane and trastuzumab. Almost 50% of the patients received prior pertuzumab. A total of 202 patients were randomized 2:1 to receive T-DM1 either alone or in combination with atezolizumab. PFS was not improved (HR = 0.82; *p* = 0.33) by the addition of atezolizumab to T-DM1, so the trial failed to meet its primary endpoint; moreover, the combination was associated with an increase in adverse events. In spite of these results, patients with PD-L1+ (using VENTANA SP142 assay) disease and TILs > 5% (by IHC) displayed a non-significant improvement of PFS (HR = 0.62 (0.34–1.13) and HR = 0·62 (0.37–1.03), respectively). Patients with CD8+ (by IHC) at the invasive margin and PD-L1+ (by gene expression: ≥ 0.307) had significant improvement in PFS (0.47 (0.25–0.85) and 0.46 (0.24–0.89)), respectively. ORR was also enhanced in PD-L1+ and TILs > 5% population compared to PD-L1 and TILs > 5% subgroups. OS data at the data cutoff is still immature although there could be an improvement in the PD-L1+ population. Hence, T-DM1 associated with atezolizumab seems to have better PFS and ORR in the PD-L1+ and TIL > 5% population compared to T-DM1 alone, but failed its primary goal in the total population [46].

The PANACEA (Keynote-014) trial is a single-arm phase Ib/II trial assessing pembrolizumab (patients with PD-L1+ tumours in a 3 + 3 dose-escalation phase Ib part; patients with both PD-L1-positive or negative at a flat dose in phase II) plus standard trastuzumab in 58 HER2+ MBC patients at progression to previous trastuzumab-based therapy. Twenty-nine% had also received prior pertuzumab and 72% had received prior T-DM1. A total of 52 patients were enrolled in the phase II stage, of which 40 had PD-L1+ disease and 12 had PD-L1- tumours. The primary endpoint of the phase Ib study was safety and recommended phase 2 dose. Pembrolizumab plus trastuzumab was safe and tolerable. The primary endpoint of the phase II study was the ORR in the PD-L1+ population, of whom 6 (15%) achieved a response. No response was seen among the PD-L1- patients. For the combined phase Ib/II PD-L1+ cohort (*n* = 46), ORR was observed in 7 (46%) patients. TILs were assessed in metastatic lesions, showing low levels in contrast to primary HER2+ breast tumours. A higher TIL count was associated with ORR and disease control (*p* = 0.006). Pembrolizumab and trastuzumab are therefore considered a safe combination showing clinical activity in PD-L1-positive, trastuzumab-resistant advanced HER2+ breast cancer [47]. 

The JAVELIN Solid Tumour trial is a phase Ib trial testing avelumab monotherapy in pretreated MBC independently of BC subtype or PD-L1 expression, in which 15.5% of patients were HER2+. The primary endpoint for this efficacy expansion cohort was confirmed best overall response, which was 3.0% for the overall population. No HER2+ patients responded to avelumab, although anti-HER2 blockade was not combined with the checkpoint inhibition. [110].

Durvalumab in combination with trastuzumab has been studied in a phase Ib trial in 15 heavily pretreated metastatic HER2+ breast cancer patients, all of whom were PD-L1 negative. The primary endpoint was the recommended phase 2 dose (RP2D). No significant clinical activity was observed among the enrolled population [111].

Notwithstanding this, nivolumab is currently being tested in combination with T-DXd in a phase 1b, open-label, multicentre, 2-part study in patients with HER2-expressing MBC or advanced urothelial cancer. Primary results were recently reported at ESMO (European Society of Medical Oncology) Breast Cancer congress 2022: ORR from the combination therapy in the HER2+ cohort was 65.6% (95% CI, 46.8–81.4%), failing to prove its primary endpoint. The authors concluded that the addition of nivolumab did not show any significant benefit over T-DXd alone [86].

In the early setting, atezolizumab is currently being assessed in addition to anti-HER2 blockade plus chemotherapy in APTneo and Impassion 050 trials. Both trials experimented with the use of anti-PD-L1 blockade as adjuvant treatment. Primary analysis from Impassion 050 was presented at the ESMO Virtual Plenaries program, and the study failed to meet its primary endpoint (pathologic complete response (pCR)) in the intention-to-treat population or PD-L1 population (*p* = 1.0 and *p* = 0.2, respectively). Following surgery, patients resumed their allocated treatment with atezolizumab versus placebo. Patients with the residual disease could receive to trastuzumab emtansine (T-DM1). Results regarding survival outcomes are awaited [109,112].

For metastatic patients, Atezolizumab is currently being tested in a randomized phase III trial against placebo in combination with paclitaxel, trastuzumab, and pertuzumab as first-line treatment (NRG-BR004 trial; NCT03199885) [113].

In addition, the ongoing AVIATOR phase 2 study is combining trastuzumab and vinorelbine with a novel 4-1BB/CD137 agonist (utomilumab) and an anti-PD-L1 (avelumab) in patients with HER2-positive MBC who progressed on prior trastuzumab and pertuzumab (NCT03414658) [114]. Included patients are randomized to receive trastuzumab plus vinorelbine, trastuzumab plus vinorelbine plus avelumab, or trastuzumab plus vinorelbine plus avelumab plus utomilumab.

Finally, whether atezolizumab has efficacy or not in HER2+ patients with brain metastases is currently being explored in a phase II study in combination with pertuzumab and high-dose trastuzumab (NCT03417544) [115].

### 4.2. AntiHER2-Vaccines

**Figure 2:**
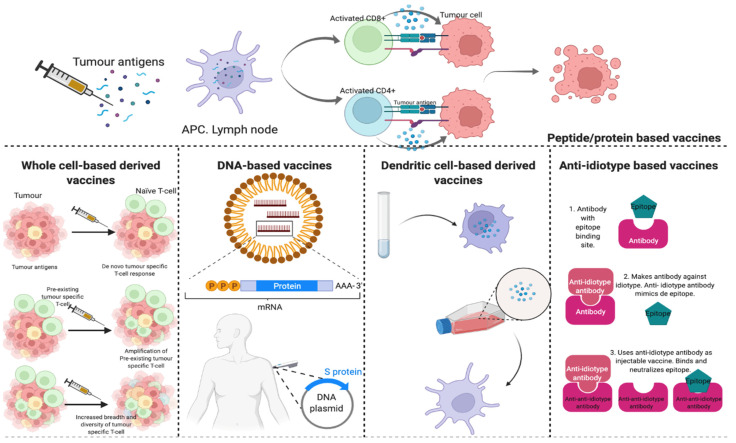
Different vaccination models harbouring HER2+ tumours. APC: Antigen-presenting cell.

Therapeutic immunization is an attractive approach to reducing the risk of recurrence among BC patients. Cancer vaccines stimulate a patient’s immune system to generate an anti-tumour response (termed type 1 immunity) by activating cytotoxic T cells, antibodies, and T helper cells. Cytotoxic CD8+ T cells recognize HER2 antigen peptides presented by MHC class I molecules, and secrete IFN- γ and TNF-α, thereby killing tumour cells. In addition, T helper cells are associated with a stronger anti-HER2 immunity response [116,117]. Figure 2 describes the different types of vaccines against HER2+ tumours and Table 3 shows the published clinical trial studying HER2 vaccines.

#### 4.2.1. HER2-Derived Peptide Vaccines

By increasing the number of epitopes, T cell-mediated tumour killing can foster T cell-mediated cytolysis and thus long-term immune memory. HER2-derived peptides include different HER2-derived molecules from extracellular, transmembrane, and intracellular domains (ECD, TMM, and ICD), such as E75 (ECD), AE37 (ICD), and the nanopeptide GP2 (TMD). B cell-derived vaccines have also been reported. Research to date has found that the E75 HER2 peptide vaccine (also known as nelipepimut-S) is presented by HLA-A2 and HLA-A3. Several early clinical and preclinical reports and have been published including a registrational phase III study [142]. Following completion of phase I and II studies, a multicentre, randomized, double-blind phase III study was designed to enroll HLA-A2+/A3+ patients with early-stage node-positive and HER2 low-expressing levels (IHC 1+/2+) to randomly receive GM-CSF either with or without E75 after completion of standard treatment. The primary objective was DFS. E75 was well tolerated but there was no difference in terms of DFS compared to placebo (*p* = 0.07); the trial was stopped for futility in 2016 according to a prespecified protocol [120].

Adjuvant trastuzumab plus concurrent E75 has been tested in two early phase clinical trials. In the first study, high-risk HER2+ BC patients were randomized to receive either trastuzumab alone or trastuzumab plus E75/GM-CSF. No disease recurrence was observed after 36 months of follow-up in the experimental group.

In the second one, E75 added to trastuzumab and GM-CSF was compared to trastuzumab plus GM-CSF alone in a randomized phase IIb trial which included HER2 low to intermediate-expressing (1+/2+) BC after completion of standard therapy. The trial also included a proportion of TNBC patients. The combination therapy did not add toxicity over trastuzumab alone, nevertheless, this combination did not show clinical benefit in the intention-to-treat population of patients with HER2+ BC (*p* = 0.18), although a significant improvement in DFS was seen in the TNBC population (*p* = 0.01) [121]. 

GP2 + GM-CSF has been assessed in the adjuvant setting in a phase IIB placebo-controlled randomized clinical trial including node-positive and high-risk node-negative HER2+ (any grade of IHC expression) BC patients after standard treatment completion.

The estimated 5-year DFS rate was statistically positive for HER2 3+ patients (n = 50; *p* = 0.0338). There was no difference in the 5-year DFS rate for HER2 1+/2+ population (n = 37; *p* = 0.9). Safety was tolerable. An ongoing phase III trial will reproduce this phase IIb study [128]. 

AE37 is another HER2-derived vaccine based on the AE36 hybrid peptide (776–790), which is derived from the intracellular portion of the HER2 protein, and the core portion of the MHC Class II invariant chain (the Ii-Key peptide) [143]. AE37 associated with GM-CSF was proven to be safe in the adjuvant setting in a dose-escalation phase I clinical trial [126].

A randomized phase II trial was subsequently conducted in 298 high-risk, any grade of HER2+ (IHC 1+ to 3+) BC patients. The 5-year DFS rate at 25 months was not improved compared to GM-CSF alone neither in the whole population (*p* = 0.7) nor in HER2+ (3+/FISH+) population (*p* = 0.45), however for TNBC population (defined as HER2 IHC 1+ to 2+/FISH-/HR-negative) there was a non-significant reduction in recurrence rate (77.7% in vaccinated patients vs. 49 % in control patients; *p* = 0.12) [125].

Besides T-cell specific response, humoral immunity has also been explored in early HER2+ BC. Active immunization with mimotopes (B-cell peptides) representing the mAbs’ binding epitopes might be an interesting strategy to activate a patient’s own anti-tumour immune response. Tobias et al. (2020) showed enhanced vaccine’s anti-tumour effect in mouse models with the combination of HER-Vaxx (which comprises 3 fused single peptides from ECD of Her-2/neu) and anti-PD-1 blockade [144,145]. 

Nordin et al. (2021) designed a chimeric antigenic peptide possessing both cytotoxic T lymphocytes (GP2) and B-cell (P4) peptide epitopes derived from HER2. This chimeric peptide (GP2-P4) was further conjugated to a carrier protein (KLH), forming a KLH-GP2-P4 conjugate. BALB/c mice received either KLH-GP2-P4 or KLH-GP2, which lacked to B-cell epitope. Mice immunized with the experimental conjugate produced more potent antigen-specific neutralizing antibodies against syngeneic TUBO cells (cancer cell line overexpressing HER2) in comparison to KLH-GP2 [146]. B-cell peptide vaccines have also been tested in a few early-phase clinical trials. A phase I study was performed on ten MBC patients with low protein overexpression of HER2 and positive HR. B-cell peptides were coupled to immunopotentiating reconstituted influenza virosomes which were used as carrier systems. The vaccine was safe and 80% developed anti-peptide antibodies [135,147]. Wiedermann et al. (2021) also recently published a phase Ib trial regarding HER-Vaxx in HER2+ metastatic gastric cancer, which proved safe and generated humoral and cellular immune response [148]. Another phase I clinical trial was conducted with a vaccine (B-Vaxx) containing two chimeric HER-2 B-cell peptide vaccines engineered to represent the trastuzumab- and pertuzumab-binding epitopes to overcome resistance to them. The vaccine also incorporates a T-cell epitope. 49 metastatic and/or recurrent solid tumours were included to receive the experimental treatment. The experimental treatment was carried out in 49 metastatic and/or recurrent solid tumours, finding the vaccine to be safe and well-tolerated. Two patients had a partial response, 14 had stable disease, and 19 had progressive disease [134].

#### 4.2.2. Protein-Derived Vaccines 

Utilizing a whole protein agent (HER2 ICD or ECD) to stimulate immune anti-HER2 response has a clear advantage over peptide-based vaccines, as it contains both HLA class I and II epitopes, thus avoiding HLA restrictions. Notwithstanding this effect, the clinical evidence is relatively small compared to peptide-based vaccines [7]. HER2 ICD was applied to 29 patients with HER2 overexpressing breast or ovarian cancer and with no evidence of disease after standard therapy. The vaccine was well-tolerated, and about 89% of the patients developed HER2 ICD-T cell immune response [149]. Combining both ICD and ECD has been explored by Curigliano et al. (2016). The vaccine, dHER2, is a recombinant fusion protein made of an ECD, a fragment of an ICD, and an immunostimulant called AS15. A phase I/II clinical trial enrolled 40 HER2+ metastatic BC patients to receive up to 18 doses of dHER2 as first or second-line treatment following response to trastuzumab-based therapy. dHER2 vaccine was considered to be safe and no cardiac events occurred. Clinical activity was observed with two objective responses and prolonged stable disease in up to 25% of the patients [139].

#### 4.2.3. Anti-Idiotype Vaccines

Immunological tolerance is a major issue in cancer immunotherapy, probably due to self-origin antigens. To surpass this obstacle, anti-idiotype (anti-Id) mAbs mimic TAAs in order to overcome immune tolerance. To date, only preliminary data have been published using scFv40 and scFv69, both human anti-Id scFv mAbs targeting trastuzumab in transgenic mice. Ladjemi et al. (2011) reported that scFv69 protected mice from developing HER2-positive breast tumours through a humoral Th-2-dependent mechanism [150]. Beyond these results, no clinical trials have been published to date.

#### 4.2.4. DNA-Based Vaccines

DNA-based vaccines are considered to induce an antitumour response in BC patients. Proof of concept is provided by the fact that TAA encoding genes can be transfected through a plasmid and then expressed in APCs. HER2 and mammaglobin-A (Mam-A) are well-known oncogenes that are overexpressed in BC cells and have been used as target antigens to develop DNA vaccines.

A phase I clinical trial was carried out in 14 Mam-A+ metastatic BC patients who received a Mam-A plasmid DNA vaccine and were compared to controls. After 6 months, 7 patients displayed an increase in Inducible T cell co-stimulator (ICOS) (hi) CD4+ T cells and a decrease in forkhead box P3 (FOXP3) CD4+ T cells. ICOS hi CD4+ T cells expressed IFN-y instead of IL-10, and were also capable of inducing lysis of human breast cancer cells expressing Mam-A protein. In clinical terms, the vaccine was safe and improved PFS rate at 6 months (53% vs. 33%; *p* = 0.011). HER2 positivity was found in 3 out of the 14 vaccinated patients [136,151].

#### 4.2.5. Dentritic Cell-Based Vaccines

DCs are the strongest modulators of the primary immune response. They are able to process exogenous and endogenous antigens and present them to CD4+ and CD8+ T cells. DCs-based vaccines are processed DC-based vaccines are processed by isolating stem cells from peripheral blood from BC patients, which are then transformed into immature DCs (iDCs). iDCs are then supplied with TAA and stimulated through a so-called ‘maturation cocktail’ (typically a combination of proinflammatory cytokines and Toll-like receptor agonists). Mature DCs are then infused back into patients wherein they will present the cancer antigens to CD4+ and CD8+ T cells [152]. A DC-based vaccine loaded with a HER2 antigen called Lapuleucel-T was assessed in a phase I clinical trial which included 19 HER2+ MBC patients. Therapy was well-tolerated and a significant specific cellular immune response was displayed. Clinical benefit was also observed [137]. Two-phase I clinical trials in the neoadjuvant setting have been published using DC-based vaccine joined to an ICD and ECD HER2 peptides. Both were safe and generated immunogenicity [130,131].

Finally, there is an ongoing phase II trial assessing the efficacy of vinorelbine and trastuzumab plus an anti-HER2 DC-based vaccine in association with GM-CSF in the HER2+ MBC population [153] (NCT00266110).

#### 4.2.6. Whole Cell-Based Derived Vaccines

Among the major challenges of non-cell-based cancer vaccines is the difficulty on finding the appropriate TAA in order to achieve the greatest immune response. Autologous tumour cell-derived vaccines (ATCVs) are an attractive strategy as these harbour a wide variety of TAAs, therefore inducing a polyclonal response. The main obstacle in this process is the difficulty in developing ATCVs. Three completed clinical trials have assessed the efficacy of ATCVs in BC patients. In the first one, 121 patients diagnosed with primary BC, MBC, and metastatic ovarian cancer were enrolled in a clinical trial in which they received an ACTV. The OS rate at 5 years was 96%, suggesting its efficacy [138]. In another phase II study, 42 BC patients were vaccinated with a multi-antigen vaccine, which included autologous and allogeneic breast cancer cells, three TAAs (CA15-3, CEA, and CA125), and low doses of GM-CSF and IL-2. The vaccine was safe and induced a significant increase in post-vaccination lymphocyte proliferative responses to all vaccine types except for the allogeneic-derived cells vaccine [154]. Finally, 37 breast cancer patients with depressed immunity (confirmed by lymphocyte blastogenesis assay before vaccination) were vaccinated in the adjuvant setting with an autologous, allogeneic whole-cell vaccine in order to evaluate the effect on host lymphocyte immunity and disease-specific survival. Patients received adjuvant therapy according to clinical practice, but importantly, none of them received trastuzumab as it was not available by the time of the study. It was observed that 10-year survival in vaccinated patients increased significantly compared to historical data [155]. 

## 5. Discussion

Despite the improvements seen with adjuvant trastuzumab in HER2+ breast cancer patients, relapses occur in around 20% of cases [156]. Metastatic patients have also experienced substantial survival benefits with dual anti-HER2 blockade combined with chemotherapy and with T-DM1, yet the progression of disease, and finally death occur in the majority of patients [2,42]. Immunotherapy has revolutionized the treatment of solid tumours by targeting the PD-1/PD-L1 axis with checkpoint inhibitors. In HER2+ breast cancer, however, results from immunotherapy trials have not been consistent. 

In the preclinical setting, there are a few molecular biomarkers that have acquired the role of predicting immunogenicity across HER2+ tumours. TILs are particularly abundant in TNBC but also in HER2+ breast cancer, and have shown to predict pCR after neoadjuvant anti-HER2 treatment. Conversely, in a retrospective study, the presence of high TILs (≥25%) in HER2+ BC with residual disease after neoadjuvant treatment was associated with worse event-free survival (EFS) [157]. Primary analysis from Impassion 050 trial has failed to meet its primary endpoint in either intention-to-treat or PD-L1 population, but no information regarding TILS was reported. The adjuvant phase III FinHER trial was the first study reporting an association between higher level of TILs and increased benefit from the addition of trastuzumab to chemotherapy in HER2+ early BC. In the neoadjuvant setting, in a planned secondary analysis of the NeoALTTO trial, the presence of TILS (>30% cutoff value) was considered to be a positive biomarker for pCR and evet-free survival (EFS). There was also a positive association between TILs and pCR in HER2+ and TNBC according to the GeparSixto phase II trial. Results are scant in the advanced setting as described in the CLEOPATRA trial. Interestingly, in the PANACEA trial lower levels of TILs were observed in the metastatic lesions, suggesting potentially significant differences in TME compared to primary HER2+ tumours. Despite these results, in metastatic TNBC patients from the phase 2 KEYNOTE-086 study assessing pembrolizumab in previously treated patients, a higher stromal TIL level was associated with responders and increased disease control rate (*p* = 0.01 for both endpoints) [158]. Authors from the PANACEA trial also acknowledge certain limitations of this study, such as the small sample size, the absence of a comparison group, and the fact that patients enrolled were heavily pretreated. Nonetheless, immune infiltration and response to ICIs are seemingly more active in the early setting for both HER2+ BC and TNBC [41]. 

The application or not of PD-L1 expression into routine clinical practice is still a matter of debate in breast cancer. In early-stage TNBC, the benefit from immunotherapy is independent of PD-L1 expression [159]. In contrast, in advanced TNBC results from Impassion 130 and Keynote-522 trials demonstrated that a PD-L1 ≥ 1 expression correlated with response to chemotherapy plus atezolizumab or pembrolizumab, respectively [141]. Results so far from the KATE2 or PANACEA trials only exhibit positive results in the PD-L1 positive population and in patients with high TIL count. In the JAVELIN trial assessing avelumab in pretreated HER2+ BC, it is noteworthy that no anti-PD-L1 blockade was combined with anti-HER2 blockade.

Pending results from exploratory analysis from the aforementioned APTneo and Impassion 050 trials will elucidate the relationship between TILs, PD-L1, and ICIs in the early HER2+ setting. Finally, Solinas et al. (2021) also suggests screening for other immune biomarkers such as Lymphocyte activation gene 3 (LAG3), T cell immunoglobulin, and mucin domain 3 (TIM3) as a new opportunity for patients not achieving pCR.

Besides checkpoint inhibitors, anti-HER2 agents and novel bispecific or conjugate antibodies are known to be able to exploit immune response, in monotherapy or in combination with other agents synergistically enhancing the immune system. ADCC is a mechanism belonging to the innate immune system by which an effector T cell lyses a target tumour cell whose membrane-surface antigens are bound to specific antibodies. Trastuzumab, margetuximab, T-DM1, and zenocotuzumab are known to enhance ADCC against tumour cells; moreover, lapatinib can also amplify this mechanism when combined with trastuzumab. 

Besides ADCC other mechanisms can be used, such as potentiating the cytotoxic T-cell effect against HER2+ tumour cells or fostering HER2 protein-immune cell interaction. In fact, 4-1BB (CD137) is an Anticalin protein providing co-stimulatory signals which thus activates CD8+ T cells, NK, and DCs. CD137+ and HER2-expressing cell interaction is mediated by cinrebafusp alfa, which has shown clinical activity in a dose escalation phase I study. Combining this agent with atezolizumab is currently being tested in HER2+ solid tumours and results are pending. Other bispecific mAB such as runimotamab (BTRC4017A) binds to both CD3-expressing T cells and HER2-expressing tumour cells, so crosslinking potentiates anti-tumour response.

Cytokines in combination with anti-HER2 agents are drugs that potentially enhance the anti-tumour response. RO6874281 is an immunocytokine consisting of an interleukin-2 variant targeting fibroblast activation protein-α, which is greatly upregulated in remodelling tissues such as tumours [160]. Its mechanism of anti-tumour effect occurs through TME remodelling, inhibiting cell migration and epithelial-mesenchymal transition. It is currently being assessed in combination with trastuzumab, but no trial assessing this dual combination with ICIs is ongoing, exposing an unmet clinical need. 

The aim of cancer vaccines in HER2+ BC is to stimulate patients’ immune systems to recognize HER2 antigen via active immunotherapy. E75 HER2-derived peptide is one of the main studied peptides designed to foster immune response in this disease. It has noteworthy activity in low and intermediate HER2+ expressors, where standard anti-HER2 agents are not proven to be effective. Unfortunately, results from the phase III trial of E75 failed to prove its efficacy in node-positive, HER2 1+/2+ expressing tumours. Krasniqi et al. (2019) remarks on the question of whether a possible combination between anti-HER2 blocking agents and anti-HER2 directed vaccines could be effective in HER2 low to intermediate HER2-expressing tumours [141]. In this context, it has been tested in combination with trastuzumab in a phase IIb trial with no clinical benefit in this population. It is important to highlight that the E75 peptide is restricted to the HLA-A2+/A3+ population, hence a limited number of patients are included in these trials. Also, the E75 vaccine appears to be short-lived and thus needs a booster dose to maintain immune response. Finally, the negative impact that might result from chemotherapy or radiotherapy before vaccination is also as yet unknown [161]. Besides E75, other HER2-derived peptide vaccines such as GP2 and A37 have also been tested with no increase in terms of DFS. In the case of A37, a non-significant benefit was shown in HER2+ -low expressing and TN tumours. 

Multi-epitope peptide vaccines enveloping cytotoxic T-lymphocytes, T-helper, and B-cell epitopes are another promising focus of research. Preclinical data suggest that stimulation of humoral immune response might be a reasonable strategy to diminish the recurrence rate in HER2+ tumours. Whole protein-derived vaccines containing ICD and ECD of HER2 have been tested in metastatic disease with discouraging results. Another interesting strategy involves DNA-based vaccines carrying a tumour-associated antigen. Mam-A is a known oncogene expressed in 40–80% of breast cancers and it is considered to be highly immunogenic. In a phase I clinical trial carried out in 14 Mam-A+ metastatic BC patients who received a Mam-A plasmid DNA vaccine, the treatment was safe and PFS was significantly better than control. Moreover, a dendritic cell-derived vaccine, such as Lapuleucel-T, has been tested in advanced, adjuvant, and neoadjuvant settings with variable results. Finally, whole-cell-derived vaccines are considered an attractive approach as they imply a wide variety of TAAs, in order to induce a polyclonal response. Its application in metastatic and early-stage BC seems safe but further investigation regarding its efficacy needs to be assessed.

Success from cancer vaccines will depend on an enhanced understanding of the TME, signaling pathways, and tumour-associated antigens, as well as vaccine formulations. Seemingly, a more advanced stage in which tumour burden is higher and tumours are more undifferentiated is a negative predictive factor of response to anti-HER2 cancer vaccines. 

As yet, cancer vaccines have not yet produced comparable results to immune checkpoint blockade, and larger future clinical trials are needed.

## 6. Conclusions

Breast cancer is a complex disease in which a myriad of variables plays a crucial role in defining the landscape of diagnosis and treatment. There is a rationale for immunotherapy in HER2+ disease, as has been shown in many preclinical studies, although its efficacy in the clinical setting has not yet been confirmed, probably pending greater understanding of the role of the TME and its components. All these biomarkers may differ radically between early to late and more heterogeneous stages of the disease; also, chemotherapy, in addition to radiotherapy and/or surgery, might also play a role by altering the TME. Further phase III clinical trials with novel conjugate antibodies and novel immunotherapeutic agents are needed in order to obtain a more comprehensive knowledge of this disease.

## Figures and Tables

**Table 1:** TILs assessment in different HER2+ trials in the adjuvant, neoadjuvant and metastatic settings.

Setting	Conducted Study	Type of Study	Population	Intervention	Primary Endpoint	TILs Assessment
ADJUVANT	FinHER Purmonen et al., 2011 [22,23]	Phase III RCT	232 early stage, operable BC HER2+ pts	9 weeks of trastuzumab or no trastuzumab in addition to chemotherapy	DDFS: benefit from adding trastuzumab to adjuvant Cht (HR = 0.32; *p* = 0.029)	Each 10% increase in TILs was associated with 13% reduction in the DDFS (HR 0.77; 95% CI 0.61–0.98, *p* = 0.02)
	NSAPB B-31 Romond et al., 2005 [24,25]	Phase III RCT	2043 early stage, operable BC HER2+ pts	Adjuvant Cht with or without trastuzumab	DFS: benefit from the addition of trastuzumab to adjuvant Cht (*p* < 0.001; stratified HR = 0.52); (95% CI, 0.45 to 0.60)	Pts with sTILs were statistically significantly associated with improved DFS (HR = 0.65, (95% CI, 0.49 to 0.86), *p* = 0.003)
	NCCTG Perez et al., 2005 [24,26]	Phase III RCT	1633 early stage, operable HER2+ pts	Adjuvant Cht with or without trastuzumab	DFS: Decrease in DFS events by 40% (HR, 0.60 (95% CI, 0.53 to 0.68); *p* < 0.001)	No association between high levels of TILS and benefit from trastuzumab therapy benefit (HR, 1.26 (95% CI, 0.50–3.17); *p* = 0.63) ^1^
	APHINITY Von Minckwitz et al., 2017 [27,28]	Phase III RCT	4805 early stage, operable HER2+ BC pts.	Adjuvant Cht plus 1 year of treatment with trastuzumab Added or not to pertuzumab	3-year iDFS rate: 94.1% vs. 93.2%.(HR = 0.81 (95% CI, 0.66 to 1.00); *p* = 0.045) in favor of pertuzumab	Higher levels of TILs associated with increased iDFS in pts treated with dual HER2 blockade. (HR 0.35 (95% CI 0.19–0.65); *p* = 0.003)
NEOADJUVANT	NEOALTTO De Azambuja et al., 2014 [29,30]	Phase III RCT	455 early stage HER2+ BC pts	Neodjuvant Trastuzumab, lapatinib, or the combination of both for 6 weeks followed by 12 week taxane + trastuzumab therapy followed by 3 cycles of fluorouracil, epirubicin, and cyclo- phosphamide after surgery	PCR (44% within the combination arm vs. 27% and 20% with trastuzumab and lapatinib, respectively) EFS: No difference between the lapatinib and trastuzumab groups (*p* = 0.81), nor the combination or trastuzumab groups (*p* = 0.33)	High TILs at diagnosis ^2^ was positively related to both pCR (nonlinear) and better EFS (linear) regardless of treatment group ^3^
	PAMELA Llombart-Cussac et al., 2017 [31,32]	Phase II RCT	151 early stage HER2+ BC pts regardless of hormone receptor	Neoadjuvant lapatinib and trastuzumab for 18 weeks	pCR prediction by HER2-enriched subtypes (41 vs. 10%; *p* = 0·0004)	TILs at day 15, but not baseline TILs, were significantly associated with pCR
	CHER-LOB guarneri et al., 2012 [33,34]	Phase II RCT	121 early stage HER2+ BC pts	Neoadjuvant chemotherapy plus trastuzumab, lapatinib, or both.	pCR from Cht plus Trastuzumab: 25%; Lapatinib: 27.2%; Trastuzumab + lapatinib: 44%	pCR rates were significantly higher in High TILs group compared to Low-TILs cases (59% vs. 27%, *p* = 0.011)
	GEPARSIXTO von Minckwitz et al., 2015 [35,36]	Phase II RCT	293 early stage locally advanced triple negative (157 pts) and HER2+ (136 pts) BC	HER2+ cohort: Neoadjuvant trastuzumab plus lapatinib with or without carboplatin	pCR from HER2+ cohort: No differential in pCR between carboplatin and no carboplatin group (45% vs. 50%; *p* = 0.58)	In HER2+ pts, pCR rates ≥ 75% were observed in LPBC phenotype (*p* = 0.006)
	NEOSPHERE Gianni et al., 2012 [37,38]	Phase II RCT	417 early stage, operable or locally advanced HER2+ BC pts	Neoadjuvant (A)Trastuzumab plus docetaxel (B) Pertuzumab, trastuzumab plus docetaxel (C) Pertuzumab and trastuzumab (D) pertuzumab plus docetaxel	pCR: 45·8% from group B compared with groups A (29%), C (16%) or D (24%)	Low-TILs group: lowest pCR rate (4.3%) Intermediate-TILs group: 26.9% -LPBC (26.7%) ^4^
	NeoLath Tokunaga et al., 2021 [39,40]	Phase II RCT	215 early stage, operable or locally advanced HER2+ BC pts	Neoadjuvant lapatinib and trastuzumab followed by either standard or prolonged course of lapatinib and trastuzumab plus weekly paclitaxel	CpCR- No difference between standard or prolonged course	NRP-1 ^6^ + TILs significantly associated with pCR (OR 1.08; (95% CI, 1.04–1.13); *p* < 0.0001)
	Meta-analysis Solina et al., 2017 [41]		Included trials: CHER-LOB, GeparQuattr, GeparQuinto, Geparsixto, NeoALTTO Total: 1256 pts			Significant association between high pre-treatment TIL levels and pCR rates (OR 2.46; (95% CI, 1.36–4.43); *p* = 0.003) ^5^
ADVANCED	CLEOPATRA Swain et al., 2020 [42,43]	Phase III RCT	808 locally recurrent, unresectable or metastatic previously untreated HER2+ patients	Docetaxel plus trastuzumab with or without pertuzumab as first-line treatment	PFS benefit from adding pertuzumab: 12.4 vs. 18.7 months (HR 0.69; 95% CI, 0.58–0.81)	Each 10% increase in TILs was related to longer OS (HR 0.89 (95% CI, 0.83–0.96); *p* = 0.0014) No significant association between TILs and PFS (*p* = 0.063).
	MA.31 Gelmon et al., 2015 [44,45]	Phase III RCT	652 locally recurrent, unresectable or metastatic previously untreated HER2+ patients	Taxane plus trastuzumab or lapatinib as first-line treatment	PFS benefit from trastuzumab vs. lapatinib: 11.3 vs. 9 months; (HR 1.37 (95% CI, 1.13–1.65); *p* = 0.001)	Low levels of pre-existing CD8+ infiltrates were related to better benefit from trastuzumab compared to lapatinib
	KATE2 Emens et al., 2020 [46]	Phase II RCT	330 HER2+ metastatic patients previously treated with trastuzumab and a taxane	T-DM1 with or without Atezolizumab	PFS. No significant benefit from the addition of Atezolizumab (*p* = 0.33)	Pts with TILs > 5% had non-significant PFS improvement (HR = 0·62; CI, 0.37–1.03)
	PANACEA Loi et al., 2019 [47]	Phase Ib/II single-arm trial	61 HER2+ metastatic patients at progression to trastuzumab-based therapy	Phase II: pembrolizumab plus trastuzumab	ORR in PD-L1 population (15%)	A higher number of TILs was associated with responders/disease control (*p* = 0.006)

BC: Breast cancer; RCT: Randomized controlled trial; Pts: Patients; DDFS: Distant disease-free survival; iDFS: invasive-disease–free survival; DFS: Disease-free survival; ORR: objective response rate; OS: Overall survival; EFS: Event-free survival; PFS: Progression-free survival; HR: Hazard ratio; (s)TILs: (stromal) tumour-infiltrating lymphocytes; CI: Confidence interval; NCCTG: North Central Cancer Treatment Group; Cht: Chemotherapy; pCR: Pathologic complete response; LPBC: Lymphocyte-predominant breast cancer; CpCR: Comprehensive complete response; NRP-1: Neuropilin-1. ^1^ 9.9% of the samples were classified as lymphocyte-predominant breast cancer. Also, only 8 disease recurrence events occurred in this group, probably underestimating the effect. ^2^ Cutoff value for high TILs was 30%. ^3^ The NeoALTTO trial is the first study where high levels of TILs are related to better outcomes regardless of anti-HER2 therapy. ^4^ Low-TILs group: <5% of stromal and tumoural TILs; LPBC: ≥50% stromal and tumoural TILs; Intermediate-TILS group = all other samples. ^5^ Greater association in patients from studies using 60% as the cutoff value to define high TILs. ^6^ Transmembrane protein highly expressed on CD3(+) CD4(+) TILs.

**Table 2:** Published trials with immune-checkpoint inhibitors in HER2+ breast cancer.

Setting	Study	Phase	Population	Intervention	Primary Endpoint	Results
Early stage	IMpassion050 [109]	III	226 high-risk HER2+ BC	Standard neoadjuvant treatment with or without Atezolizumab	pCR	No significant benefit from the addition of Atezolizumab (*p* = 1.0) in the ITT population nor in PD-L1+ population
Metastatic disease	KATE2 [46]	Phase II RCT	330 HER2+ metastatic patients previously treated with trastuzumab and a taxane	T-DM1 with or without Atezolizumab	PFS	No significant benefit from the addition of Atezolizumab (*p* = 0.33)
	PANACEA [47]	Single arm phase Ib/II trial	61 HER2+ metastatic patients at progression to trastuzumab-based therapy	Phase II: pembrolizumab plus trastuzumab	ORR in PD-L1+ population	6 PD-L1-positive patients achieved an objective response (15%, 90% CI 7–29)
	JAVELIN Solid Tumour trial [110]	Phase Ib	168 MBC pts independently of subtype (15.5% HER2+)	Avelumab monotherapy	Confirmed BOR	3% overall. No HER2+ pts responding to Avelumab ^1^
	CCTG IND.229 [111]	Phase Ib	14 HER2+ MBC pts previously treated with CT and anti-HER2 blockade	Durvalumab and trastuzumab	Safety and efficacy	No responses (SD in 29%)
	DS8201-A-U105 [83]	Phase Ib	48 pts (32 HER2+ pts-cohort 1 and 16 HER2 low-cohort 2) ^2^	Nivolumab and T-Dxt	confirmed ORR	No significant benefit. ORR: 65.6% (95% CI, 46.8–81.4) for cohort 1 50% (95% CI, 24.7–75.3) for cohort 2.

BC: Breast cancer; pCR: Pathological complete response; ITT: Intention-to-treat population; PFS: Progression-free survival; MBC: Metastatic breast cancer; CT: Chemotherapy; BOR: Best overall response; ORR: overall response rate; IHC: Immunochemistry; FISH: Fluorescence in situ hybridization; T-Dxt: Trastuzumab Deruxtecan; ORR: Overall response rate. SD: Stable disease; Pts: patients. ^1^ In this trial anti-HER2 blockade is not combined with the checkpoint inhibition. ^2^ HER2-low described as IHC 1+ or 2+ FISH negative.

**Table 3:** Published clinical trials with HER2+ breast cancer patients treated with anti-HER2 vaccines.

PUBLISHED						
Setting		Author	Drug	Nº pts	Phase	Outcomes
**Early**	**Adjuvant**	Benavides et al., 2009 [118]	E75	150	II	Immune response (*p* = 0.02) DFS (*p* = 0.4 low expressors; *p* = 0.7 overexpressors) Mortality (*p* = 0.08 low expressors LE; *p* = 0.6 overexpressors)
		Peoples et al., 2005 [119]	E75	53	I	DFS (*p* < 0.19)
		Mittendorf et al., 2019 [120]	E75	758	III	DFS (*p* = 0.07)
		Clifton et al. (2020) [121]	E75	275 (HER2 IHC 1+/2+, nonamplified FISH)	IIb	24-month DFS (*p* = 0.18)
		Peoples et al., 2008; Mittendorf et al., 2012 [122,123]	E75	186	I/II	Immunological response DFS (*p* = 0.04 20 months; *p* = 0.08 24 months)
		Patil et al., 2010 [124]	E75	52	I/II	Immunological response Safety
		Mittendorf et al., 2016 [125]	AE37	144 HER2+ (3+/positive FISH)	II	DFS (*p* = 0.45) Immunological response
		Holmes et al.2008 [126]	AE37	15	I	Immune response
		Mittendorf et al., 2016 [125]	GP2	101	II	DFS (*p* = 0.43)
		Clifton et al.2017 [127]	GP2	17	I	Immunological response
		Patel et al., 2021 [128]	GP2	168 (96 HER2+ 3+ After T; 72 HER2+ 1+/2+)	IIb	DFS: HER 3+: *p* = 0.03388 HER2 1+/2+: *p* = 0.41
		Valdes-Zayas et al., 2017 [129]	Anti-ganglyoside vaccine (NeuGcGM3)	22	III	Immunological response
	**Neoadjuvant**	Koski et al., 2012 [130]	DC-based vaccine	27	I	Immunological response
		Lowenfeld et al., 2017 [131]	DC-based vaccine	54	I	Immunological response -pCR
		Higgins et al., 2017 [132]	Wilms’ tumor 1 immunotherapeutic	15	I	Safety Immunological response
		Czerniecki et al., 2007 [133]	DC-based vaccine	13	I	Immunological response
**Metastatic**		Bekaii-Saab et al., 2019 [134]	Chimeric HER-2 B-cell peptide vaccines + T-cell epitope	49 (solid tumours)	I	Safety immunogenicity
		Wiedermann et al., 2010 [135]	3 HER2-peptides plus influenza virosome	10 (HER2+ 1+/2+ FISH negative)	I	Safety immunogenicity
		Tiriveedhi et al., 2014 [136]	Mam-A plasmid DNA	14	I	Safety Immunogenicity Efficacy: PFS improvement at 6 months (*p* = 0.011)
		Park et al., 2007 [137]	DC-based vaccine	19	I	Safety Immunogenicity
		Ahlert et al. [138]	ATCV	121 (of which 27 were MBC)	NR	Immunogenicity Efficacy. NS Survival benefit (*p* = 0.18)
		Curigliano et al., 2016 [139]	Protein-derived (ECD+ICD + AS15) vaccine: dHER2	40	I/II	Safety Response rate (2/25 PR), prolonged SD in 25%
		Disis et al., 2009 [140]	Protein-derived T-cell vaccine	22	I/II	Safety and immunogenicity

Modified from Krasniqi et al., 2019 [141]. Pts: Patients; pCR: Pathological complete response; DFS: disease-free survival; ORR: Overall response rate; DC: Dendritic cell; ATCV: Autologous tumour cells-derived vaccine; MBC: Metastatic breast cancer; NR: Not reported; NS: Non-significant; ECD: Extracellular domain; ICD: Intra cellular domain; SD: Stable disease; PR: Partial response; DCIS: Ductal in situ carcinoma; FISH: Fluorescence in situ hybridization.
